# Truth of Floating Carotid Plaques

**DOI:** 10.3389/fneur.2017.00673

**Published:** 2017-12-12

**Authors:** Wei Liu, Shuo Lu, Yibo Feng, Zhiyong Zhang, Peng Liu, Zunjing Liu

**Affiliations:** ^1^Department of Neurology, China-Japan Friendship Hospital, Beijing, China; ^2^Department of Cardiovascular Surgery, China-Japan Friendship Hospital, Beijing, China

**Keywords:** floating carotid plaque, ultrasonography, carotid endarterectomy, atherosclerosis, pathological features, ischemic stroke

## Abstract

The floating plaque in carotid artery is an uncommon condition that can be detected by a duplex ultrasonography scan and is a high-risk factor for embolic cerebrovascular disease. The histopathological features of floating plaque in carotid artery vary. To the best of our knowledge, there is still considerable controversy about the treatment of floating carotid plaque. In this case, the floating carotid plaque was located in the edge of atherosclerotic plaque in common carotid artery, pathological finding following carotid endarterectomy confirmed that the mobile substances were formed by the contents of the plaque protruding into the carotid lumen after the rupture of the fibrous cap, without mural thrombus. This pathological change was different from those of the mobile substances, which were commonly considered as mural thrombotic substances of ulcer plaque caused by the ruptures of fibrous cap of vulnerable plaque. According to pathological differences, we investigated pathogenesis of ischemic cerebrovascular disease caused by floating carotid plaque and possible treatments.

## Introduction

The floating plaques in carotid artery are mobile substances attached to the vessel wall and have reciprocating exercise along with the cardiac cycle detected by ultrasonography. These floating plaques are significantly associated with transient ischemic attack and progressive stroke. Previous reports believed that the pathological features of the floating plaque were mural thrombotic substances of ulcer plaque caused by the ruptures of fibrous cap of vulnerable plaque (1–4). Some cases reported that the floating plaques could disappear after antithrombotic therapy (anticoagulant or antiplatelet therapy) (2, 5, 6). Other cases showed that although giving anticoagulant therapy, the mobile plaque still progressed and led to recurrent ischemic events in a short time, but there was no pathological evidence of floating plaque (3, 4, 7). We combined this case with those from literature review to discuss the pathogenesis of ischemic cerebrovascular disease caused by floating carotid plaque and possibly treatments, to provide a theoretical basis for the diagnosis and treatment of this disease.

## Case Report

**Figure 1:**
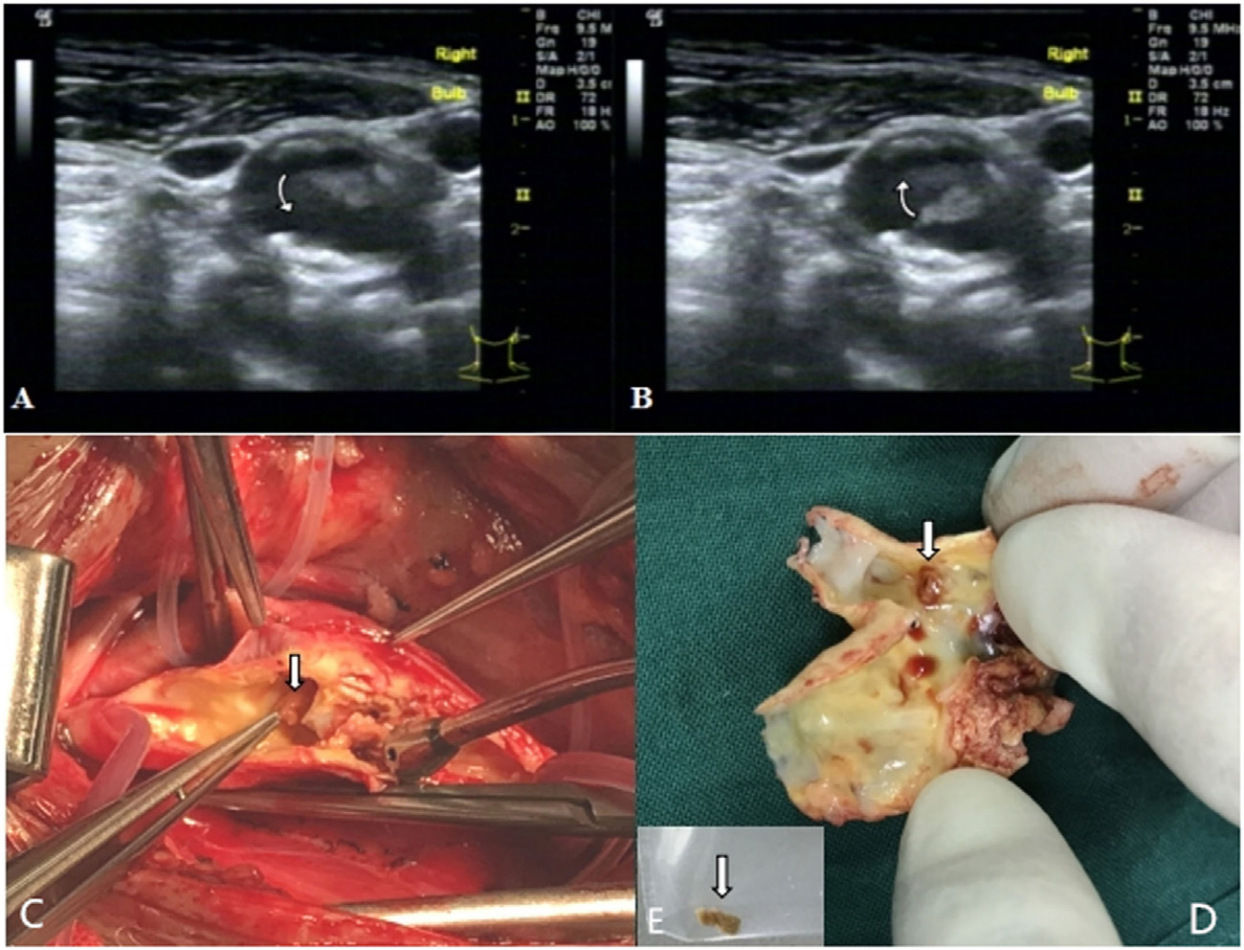
The two-dimensional ultrasonography of right carotid artery shows that the floating carotid plaques in the medial wall of the bifurcation of the distal carotid artery, moving with the cardiac cycle [**(A,B)** arrows]. The floating material with pedicel can be found in the bifurcation of carotid artery during operation [arrows **(C)**]. The floating material [arrow **(E)**] was stripped off from the base of the desquamated plaque [arrow **(D)**].

A 59-year-old man was admitted to our institution due to dizziness and right tinnitus. Previous history was notable for hypertension, hyperhomocysteinemia, and coronary atherosclerotic disease, and the patient had received coronary artery bypass graft surgery. He took aspirin 100 mg/day and anti-hypertensive medications regularly. No obvious abnormality was found in head CT. The carotid ultrasound (GE LOGIQ9 duplex sonography with a 10L transducer) showed the endomembrane of bilateral carotid arteries thickening with multiple plaques formation; multiple plaques with heterogeneous echotexture in the distal segment of right common carotid artery with about 90% reduction in diameter; and the about 0.7 cm × 0.3 cm pedicle substances with homogeneous echotexture in the medial wall of the bifurcation of the distal carotid artery, moving with cardiac cycle (free-floating plaques) (Figures 1A,B).

**Figure 2:**
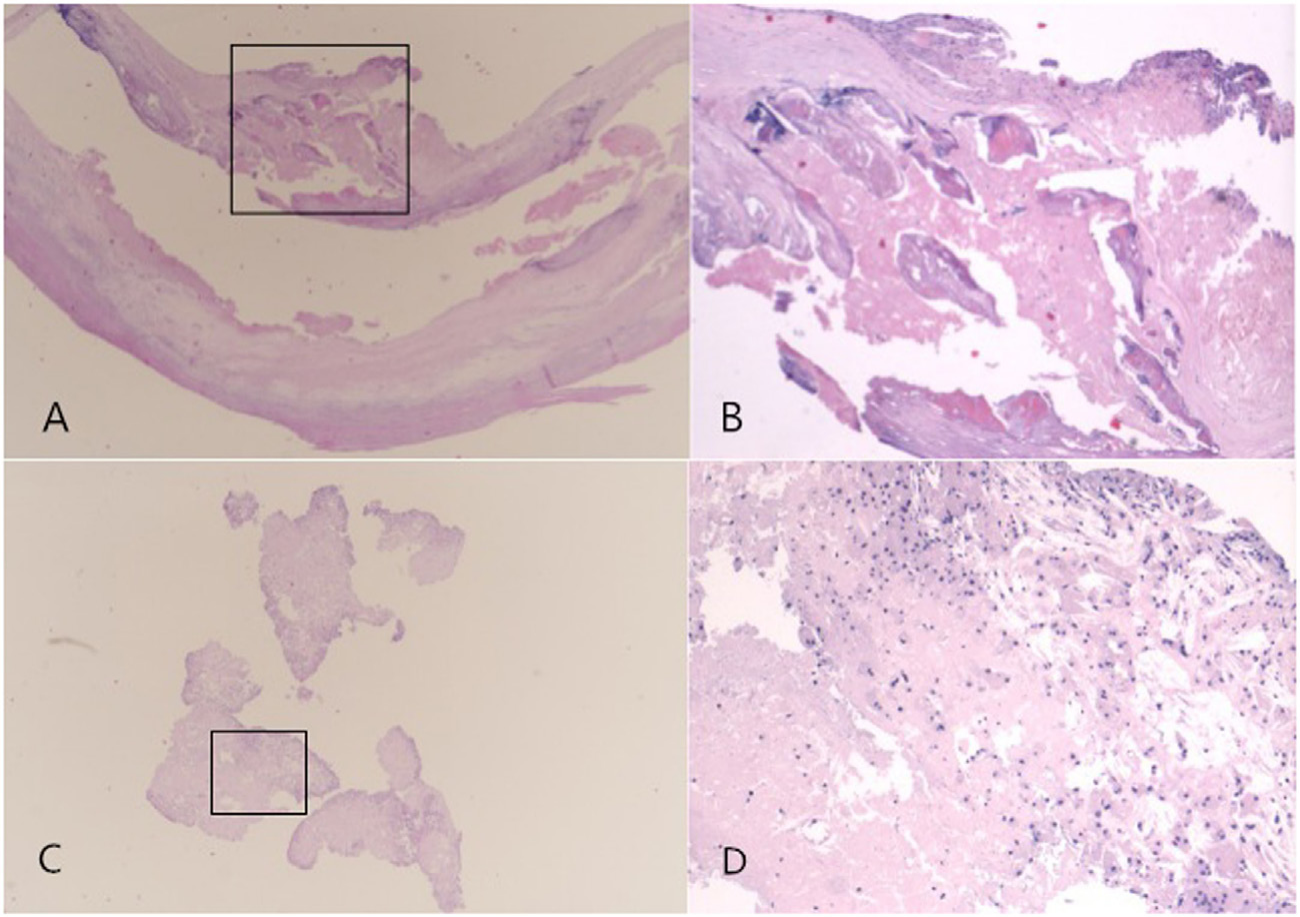
Pathological examination: the fibrous cap in the base of floating plaque was incomplete, in which lipid necrosis and calcification can be found, and tissue cells which ingested the lipids were protrudes from the fibrous cap [HE staining; original magnification 2× for **(A)**, and 4× for **(B)**]. The floating plaque was consisted of tissue cells ingesting the lipids and cholesterol clefts, there was lipid necrosis inside it [HE staining; original magnification 2× for **(C)**, and 10× for **(D)**].

Considering unstable plaques in the right carotid artery is a high-risk factor for further vascular events, we performed the cardiovascular surgery immediately. The patient was treated with carotid endarterectomy under general anesthesia. During operation, we found atherosclerotic plaques in common carotid artery and the bifurcation of carotid artery which caused stenosis, a white, fragile, and brittle floating plaque with pedicel could be found in the bifurcation of carotid artery, with a size of about 0.7 cm × 0.3 cm (Figures 1C–E). Pathological examination revealed that the fibrous cap in the base of floating plaque was incomplete, in which lipid necrosis and calcification could be found, and tissue cells ingesting the lipids protruded from the fibrous cap (Figures 2A,B). The floating plaque consisted of tissue cells ingesting the lipids and cholesterol clefts, inside of lipid necrosis (Figures 2C,D). The patient passed the operation smoothly without any accidents and complications. There were no vascular events during a 6-month follow-up period.

## Discussion

The cases about the mobile substances in carotid artery can be traced back as early as the 1860s (5). This phenomenon is relatively rare in clinical practice, but because of its instability, easily causing recurrent arterial embolism events in the carotid artery supply area, early clinical intervention is necessary (1–8). Duplex ultrasonography scan is a good method to assess and detect carotid mobile lesions, with high sensitivity and specificity, and can detect the bloodstream of carotid artery and the shape of carotid plaque in real time (8, 9). However, there are few studies about the pathogenesis and histopathological features of carotid artery mobile lesions, and its histopathological features vary, so there is still considerable controversy about the treatment of floating carotid plaque. Carotid mobile lesions may be caused by thrombotic substances coming from heart, intimal flap of carotid dissection, unstable atherosclerotic plaque, and its mural thrombus. The studies of Kimura and Uchino and Schlachetzki et al. revealed that cardiogenic embolism might be the reason of mobile carotid thrombus, all their patients had cardioembolic stroke (atrial fibrillation, acute myocardial infarction, and atrial septum aneurysm), and no underlying wall defect was found during operation (10, 11). Other reports believed that carotid artery dissection was also one reason for it (5). The reports of Stewart et al. and Nakajima et al. considered that the histopathology of the floating plaque was mural thrombotic substances of ulcer plaque caused by the ruptures of fibrous cap of vulnerable plaque, not fibrin-rich thrombus (1, 4). Therefore, carotid mobile substances may be formed by *in situ* thrombosis in ulcer plaque.

Floating plaque in carotid artery is one type of carotid artery mobile lesions. In this case, ultrasound examination found atherosclerotic plaques and stenosis in common carotid artery and the pedicle substances with homogeneous echotexture in the medial wall of the bifurcation of the distal carotid artery, moving with cardiac cycle. Regarding the phenomenon of carotid artery mobile materials caused by plaques, Funaki et al. classified carotid artery floating plaques into four groups based on the ultrasonographic features of 18 patients with carotid mobile plaques (9): ① Mobile components that were localized at the surface of the plaque and that rose and fell in a manner inconsistent with or exceeding arterial pulsatile wall motion (jellyfish-sign); ② mobile components inside the plaque that changed slowly and irregularly liked viscous liquid (liquefaction-sign); ③ movements localized within an ulcer’s inner surface; ④ movements of protuberances. But, this classification did not provide the corresponding pathological features. In our case, mobile carotid atheromatous materials was found in the distal end of narrowest area in carotid artery. The fibrous cap in the base of floating plaque was incomplete, in which lipid necrosis and calcification could be found, and tissue cells ingesting the lipids protruded from the fibrous cap. The floating plaque consisted of tissue cells ingesting the lipids and cholesterol clefts, inside of lipid necrosis but without thrombus materials. According to histopathological features, we considered that this floating plaque with thin and incomplete fibrous cap was formed by lipid necrotic core protuberating in vascular lumen, becoming an endovascular mobile material. At the same time, we noticed that the stenosis of carotid artery with floating plaque was not severity based on the feature of stripped plaque. This was consistent with the results of previous studies that part of fibrous capsule of plaque was easy to be broken, because the cap margin or shoulder region is often the weakest spot (12).

Our case found that the histopathological features of floating plaque were different from previously reported features. The previous cases considered that the histopathology of the floating plaque was mural thrombotic substances of ulcer plaque caused by the ruptures of fibrous cap of vulnerable plaque. Subsequently, the loose mural thrombus dropped off and led to clinical thrombotic events (1–4). In this case, the floating plaque contents were protuberated in vascular lumen and became an endovascular mobile material, probably leading to clinical embolic events.

Therefore, the different pathological features of carotid plaque are the main reason for the controversy about the treatment of floating carotid plaque. The investigators could observe the plaque disappeared or dissolved in patients with mural thrombotic plaques after anticoagulant or antithrombotic therapy, and no clinical ischemic events occurred (2, 5, 6). But in this kind of floating plaques, anticoagulant or antiplatelet therapy was not effective, because the floating plaque was formed by plaque’s contents, not a thrombotic substance (of course, antithrombotic therapy may reduce thrombosis on the surface of the floating plaque). It also can explain why some mobile plaques still progressed and expanded, leading to recurrent ischemic events in a short time in spite of anticoagulant therapy (3, 7, 13). However, the existing clinical techniques are difficult to distinguish the components of different floating plaques. For the treatment of carotid floating plaques, resecting (carotid endarterectomy) or covering (carotid artery stenting) may be beneficial in reducing the incidence of clinical ischemia events (1, 3, 5, 14, 15). But, carotid artery stenting may increase the risk of embolic events, so endarterectomy may be the best choice. But the effect of carotid endarterectomy for this special change will be studies in the future based on the studies with a large sample size. Of course, with the development of imaging technology, the component of floating plaque could be identified using the advanced imaging techniques such as high-resolution NMR technique, which could provide an important direction for the selection of clinical interventions (16).

## Conclusion

Carotid arterial plaque is a risk factor for ischemic events, but its pathogenesis varies. When the pathological nature of the active plaque is not confirmed, maybe CEA surgery is a relatively reasonable treatment.

## Declaration of Patient Consent

The authors certify that they have obtained all appropriate patient consent forms. In the form, the patient has given his consent for his images and other clinical information to be reported in the journal. The patient understands that his name and initial will not be published and due efforts will be made to conceal his identity, but anonymity cannot be guaranteed.

## Author Contributions

ZL designed this study. YF and ZZ searched database. WL and SL extracted and analyzed. WL and SL wrote the article. ZZ and PL made critical revision of the article. ZL final approval of the version to be published.

## Conflict of Interest Statement

The authors declare that the research was conducted in the absence of any commercial or financial relationships that could be construed as a potential conflict of interest.
